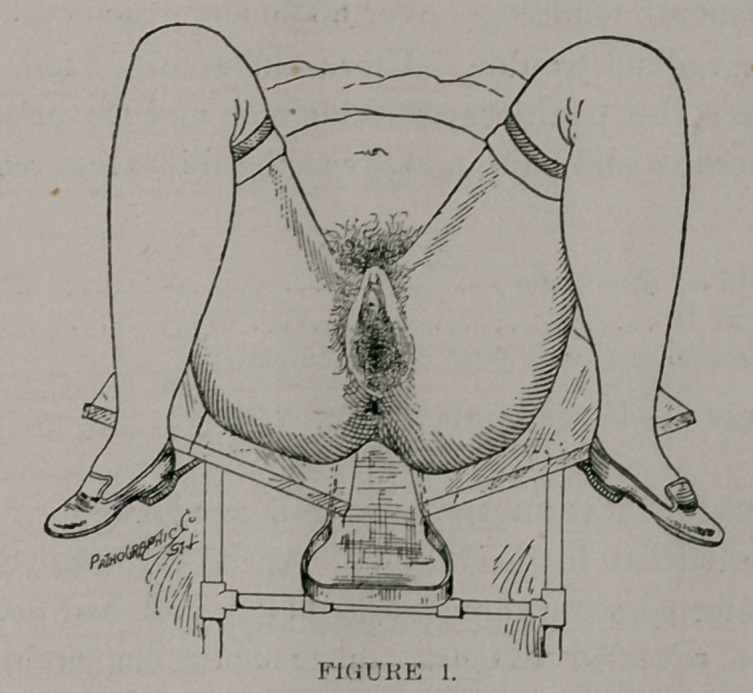# Chancroid of Eight Years’ Standing

**Published:** 1894-09

**Authors:** R. M. Kirley

**Affiliations:** St. Louis; Superintendent of St. Louis Female Hospital


### CHANCROID OF EIGHT YEARS' STANDING.

By R. M. KIRLEY, M.D.,
Superintendent of St. Louis Female Hospital

FEMALE HOSPITAL CASE.—L. O'B., aged thirty-nine; nativity, Mississippi; occupation, prostitute; was admitted into Female Hospital in March, 1894.

FIGURE 1.

Family history could not be obtained. She was suffering from severe vaginal inflammation and chancroids on posterior vaginal wall from cervix to labia, underlaid with deep scar tissue. She states that she contracted chancroids eight years ago and has had them ever since. She has no history or evidence of syphilis, and the deep scar tissue indicated that the chancroids have maintained their locality.

## Figures and Tables

**FIGURE 1. f1:**